# Substance use disorder recovery research opportunities: perspectives from a federal interagency workgroup

**DOI:** 10.3389/fpubh.2025.1585533

**Published:** 2025-04-10

**Authors:** Peter Gaumond, Lindsey Ann Martin, Brett T. Hagman, Mary J. Davis

**Affiliations:** ^1^Office of National Drug Control Policy, Executive Office of the President, Washington, DC, United States; ^2^National Institute on Drug Abuse, National Institutes of Health, North Bethesda, MD, United States; ^3^National Institute on Alcohol Abuse and Alcoholism, National Institutes of Health, Bethesda, MD, United States

**Keywords:** recovery, remission, substance use disorder, recovery capital, peer recovery support services

## Abstract

In 2024, the Office of National Drug Control Policy (ONDCP) convened an interagency working group (IWG) comprised of 30 federal agencies to identify federal recovery research gaps and opportunities on recovery from substance use disorder (SUD). This article outlines the process undertaken to identify these research opportunities and describes four core research topic areas and three cross-cutting themes and provides the rationale for their selection. It also identifies potential pathways for recovery research, including evaluation and data collection activities, and discusses challenges and potential opportunities for recovery research.

## Introduction

Research on SUD recovery has grown rapidly over the past few years. Nonetheless, recovery science lags far behind science on topics such as the treatment of substance use disorder and addiction-related pharmacotherapies. Moreover, research has tended to focus on specialty treatment populations, which represent a relatively small subset of individuals who have resolved alcohol or other drug (AOD) problems. Among individuals who reported having resolved an AOD problem, Kelly and colleagues found that 27.6 percent had received specialty treatment while 21.8 percent had received recovery support, and 45.1 percent had participated in mutual aid groups (1). Similarly, in 2023, while an estimated 54.2 million U.S. individuals age 12 and over needed SUD treatment, less than one quarter (12.8 million) of these individuals received treatment (2). A significant portion of individuals experiencing AOD problems report natural recovery, the resolution of an AOD problem without treatment, mutual aid, or other services or supports. To more fully understand the addiction, recovery, and remission processes, research needs to be broadened to include those for whom the trajectory to recovery or remission does not include formal treatment.

The 2022 National Drug Control Strategy (NDCS) noted the need for “targeted, actionable research to guide policy and resource allocation decisions in the recovery domain” and called for the establishment of an interagency workgroup (IWG) to advance such an effort, directing federal agencies to: (1) summarize the current scientific knowledge of the recovery process and recovery support services (RSS); (2) catalog current federally-funded research and evaluation efforts germane to these topics; and (3) identify key areas where additional research is needed. While the NDCS called on 16 federal agencies to participate in the IWG, a total of 30, including six components of the Department of Health and Human Services, participated. Supplementary material 2 provides a list of participating federal agencies.

To establish a baseline of current knowledge, ONDCP staff consulted with IWG participants and compiled existing literature reviews and research publications. IWG participants gathered information on recent, current, and planned federally supported research, evaluation, and data collection efforts as well as services, billing and other program data. In addition, participating agencies were canvassed to identify recovery-related research, evaluation, and data collection activities in which they were not currently engaged, but which were possible under agencies’ statutory authorities (See Supplementary material 1 for the table used to collect information on activities within agencies’ purview and scope, whether they are currently engaged in them or not). Following a review and discussion of the current agency efforts and of the scientific knowledge base, IWG participants developed a list of research opportunity areas. To be considered a research opportunity, research topics need to represent an underdeveloped area of research.

IWG participants identified seven research opportunity areas, including four core research topics and three cross-cutting themes. The core topics were recovery support services (RSS); organization and financing of systems and services; recovery and remission trajectories and intervention points; and recovery measure validation. The three cross-cutting themes were: role of people with lived and living experience (PWLLE) of SUD in policy, systems, services and research; ecosystems (i.e., multilevel approaches to research); and stigma.

**Figure 1:**
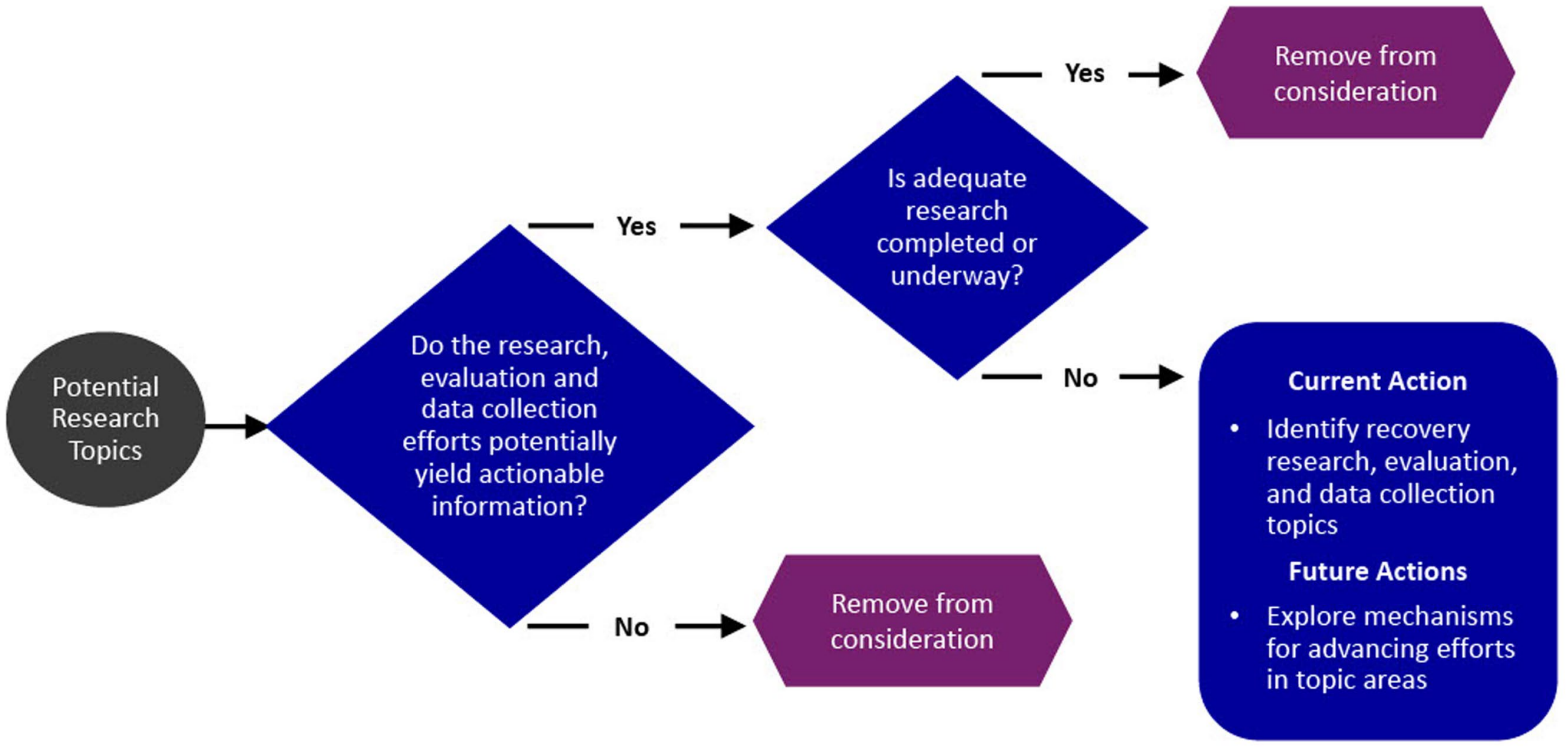
Federal recovery research interagency working group process.

Figure 1 depicts the working group’s process for identifying the recovery research opportunity areas.

### Recovery research opportunity areas

In the following section we describe the scope of the identified research opportunities and provide the rationale for their selection. Most participating federal agencies reported research, evaluation, and data collection activities focused on recovery support services (*n* = 25). This was followed by fewer agency activities related to organization and financing of systems/services (*n* = 13), recovery and remission trajectories and intervention points (*n* = 6), and measure validation (*n* = 4). In terms of cross-cutting themes, agencies reported fewer activities related to the role of people with lived and living experience in SUD (*n* = 6), ecosystems (*n* = 2), and stigma (*n* = 5). In Table 1, we provide a list of sample topics/questions by research area.

**Table 1:** Recovery research opportunity areas.

Core research areas	Potential research topics (not an exhaustive list)
Recovery support services	Efficacy and effectiveness research on recovery support services including cost-effectiveness evaluations Provision of recovery support services and the role of peer-led organizations Use of mobile health and other digital technologies as forms of recovery support and mechanisms for continuing engagement in services, including harm reduction and treatment Studies examining recovery support in a variety of different contexts and settings, including community settings, secondary and higher education settings, workplaces, specialty SUD treatment, emergency departments and other hospital settings, primary care, courts, and other clinical and non-clinical settings Access to recovery support services in underserved communities Studies focused on the tailoring of recovery support services to meet the needs of women, adolescents/young adults, older adults, and other groups
Organization and financing of systems/services	Studies on current or potential funding pathways for recovery support services Studies on the role of the organizational home (e.g., peer-led organization, specialty SUD treatment provider, hospital emergency department, county government, etc.) of peer support workers in ensuring access, quality, comprehensiveness, coordination, and continuity of services Studies on financial/reimbursement models that support the integration of PWLLE in the healthcare system (e.g., primary care, emergency department, etc.) Research developing educational interventions to educate states, treatment providers, and service providers about funding sources currently available to fund RSS Research that evaluates statutory and regulatory policy changes that provide greater authority and flexibility to provide RSS Research comparing current or potential funding mechanisms for recovery support services
Recovery and remission trajectories and intervention points	The identification of mechanisms of behavior change (i.e., social, psychological, neurobiological) to characterize both initiation and maintenance of behavior change process in recovery Role of co-occurring health conditions such as mental health conditions and chronic pain in recovery and remission over time Research to better understand how aspects of wellbeing, quality of life, and biopsychosocial functioning affect recovery and how these factors serve as important outcomes in evaluating recovery trajectories Research to identify, characterize, and validate phases of recovery to understand more about how to track episodes of use or returns to ongoing use, identify changes in clinical symptoms and functioning, and identify benchmarks on which to judge when recovery has occurred Research on community-level factors, including the relationship of the social determinants of health to the likelihood, prevalence, and trajectories Characterizing recovery outcomes and behavior change pathways between those who seek formal treatment and those who in engage in “natural recovery”
Recovery measure validation	Studies examining the reliability and validity of existing recovery measures across populations Studies on the theoretical constructs underlying how recovery is defined and varies across populations Research seeking to integrate measurement-based care and to evaluate recovery metrics in clinical practice

Cross-cutting themes	Potential approaches to enhance recovery research
Role of PWLLE of SUD in policy, systems, services and research	Community/partner engaged research approaches that include PWLLE as equal partners in the research process Evaluating the impact of PWLLE in research, including the uptake of research findings Examining the role of PWLLE in reducing stigma around SUD among policymakers, systems administrators, health care providers, and community members Developing and testing strategies to engage PWLLE in research, services, and policy
Ecosystems (multi-level) research approaches	Research approaches that are multi-level, with an emphasis on community and organizational-level factors associated with improvements in recovery initiation, recovery prevalence, and recovery duration Community, workplace, or social networks associated with increased likelihood of recovery Strategies for building recovery capital at the community or organizational level
Stigma	Research approaches that focus on the various types of stigma (i.e., social, structural, and self-stigma) and its impact on SUD help-seeking, access to and effectiveness of care, and community support for and adoption of policies that support recovery Development and testing strategies to reduce stigma in specialty treatment, general healthcare, and community settings Examining the intersection of substance use stigma and other factors, including justice involvement Use of non-stigmatizing language in research and dissemination activities

## Rationale for inclusion—core recovery research areas

### Recovery support services

Recovery support services (RSS) are non-clinical services that help individuals initiate and sustain recovery from SUD. They can be offered in conjunction with treatment or other clinical services or separately, through entities such as recovery community centers, recovery residences, recovery community organizations, recovery high schools, collegiate recovery programs, and other organizations. The majority of RSS are offered by PWLLE, or “peers.” RSS provided by PWLLE are known as peer recovery support services (PRSS). During the final decade of the 20th century, PRSS began to be embedded in clinical service systems and grew to be recognized as a separate form of community-based services and support infrastructure. This was due, in no small measure, to the Recovery Community Services Program, a Substance Abuse and Mental Health Services Administration (SAMHSA) grant program that continues to this day. The Recovery Community Services Program has played a key role in the development of recovery community organizations and recovery community centers nationally. Recovery housing, which had spread nationally by the middle of the 20th century (3), is the most studied form of peer recovery support services. Research on other forms, including recovery coaching, collegiate recovery programs, recovery high schools (3), and recovery community organizations (4) is emerging, but is far less developed. The role and impact of mutual aid groups, such as Alcoholics Anonymous, Narcotics Anonymous, and SMART Recovery, is an additional gap area.

### Organization and financing of systems and services

While there is growing evidence that PRSS can be critical to initiating and maintaining recovery from SUD (5–7), organizational and financing barriers remain. For example, a recent study found that peer support services covered by Medicaid were underutilized by people with opioid use disorder (8). PRSS are typically funded through federal, state, and local sources, including Medicaid; Substance Use Prevention, Treatment, and Recovery Services Block Grant; state general revenue; federal discretionary grants; and drug courts. Private health insurance, local fundraising efforts, and philanthropies also play a role. PRSS do not have dedicated federal funding streams such as those provided for primary prevention and treatment. Because PRSS are often offered by non-traditional providers, states, local governments, and private insurers may not be accustomed to purchasing services from such entities and may not have standards in place for doing so. Multi-state public and private payers may also be impeded by inconsistencies in peer certification criteria and differing regulatory requirements across states. Questions remain about the best approaches for integrating PRSS and clinical services and how they may build community-level recovery capital in a manner that reaches non-treatment populations.

### Recovery and remission trajectories and intervention points

The recovery domain is anchored in SAMHSA’s working definition of recovery as “a process of change through which individuals improve their health and wellness, live a self-directed life, and strive to reach their full potential.” Under this definition, recovery has four major dimensions: health, home, purpose, and community (9). Recovery is viewed through a strengths-based lens with a focus on improvements in health, social functioning, and quality of life. Importantly, multiple stakeholders note the heterogeneity of recovery pathways, arguing that a range of potential intervention points will need to be identified, studied and validated. For example, in the development of the National Institute on Alcohol Abuse and Alcoholism definition of recovery, recovery stakeholders identified and acknowledged that recovery involves multiple domains and is often marked by the fulfillment of basic needs, enhancements in social support and spiritualty, and improvements in physical and mental health, quality of life, and other dimensions of wellbeing (10). In addition, participants in a recent National Institutes of Health HEAL Initiative recovery workshop also noted how individual recovery trajectories and narratives vary widely, and how the recovery journey can be impacted by challenges such as lack of employment and treatment access (11). From a research perspective, recovery from SUD is commonly characterized as a process of initiation and maintenance of health behavior change that can include periods of remission and recurrence (12, 13). Understanding the heterogenous behavior change pathways and trajectories of recovery from SUD is critical for characterizing phases of recovery and identifying important intervention points for initiating and/or sustaining recovery and better informing federal policies that support the recovery process.

### Recovery measure validation

Recovery measure validation was not initially identified as a core research opportunity area given the range of validated recovery capital and quality of life measures, such as the Brief Assessment of Recovery Capital or BARC-10, and other relevant validated measures. However, additional reliability and validity testing is needed to better understand contextual variations of recovery capital (14). Addiction recovery stakeholders and researchers clearly acknowledge that wellbeing and biopsychosocial functioning constructs are considered integral in defining recovery. Additional research needs to address how best to operationalize these constructs, identify threshold values to mark clinically meaningful progress, and identify the constructs that are most integral to success in recovery from an alcohol or other drug use disorder (10, 11). More research is also needed to support recovery-focused measurement-based care, a process that utilizes standardized, valid, repeated measurements to track an individual’s progress over time (15).

## Three cross-cutting themes to enhance recovery research

### Role of people with lived and living experience of SUD

PWLLE bring experience and perspective to research, helping ensure that study designs are well-tailored to targeted communities. Additionally, when leading research or taking part in community-level data collection, PWLLE can overcome common barriers to participation, including trust of the researchers. For this reason, federal agencies have taken significant steps to engage PWLLE in the planning, implementation and dissemination of research. The NIH HEAL Initiative’s Patient and Community Engagement Resources webpage is one example. Working groups and panels comprised of PWLLE provide another example of how federal agencies engage people affected by SUD in their response efforts. There is a critical need to increase the number of researchers with lived experience leading federally supported research. This brings an important perspective to research design, can foster greater trust between the research team and community partners, and can lead to greater acceptability and sustainability of studied evidence-based interventions (16). Engagement of PWLLE in the development of programs that impact them has been limited. Future work could follow the model adopted by Health Canada that includes a PWLLE Council and extensive involvement of PWLLE in research through the Canadian Research Initiative in Substance Misuse (CRISM).

### Ecosystems (multi-level) research approaches

People with SUD and those in recovery are embedded in and interact with multiple ecosystems. These include family, neighborhood, community, school, work, and faith groups. They also include formal systems, such as specialty SUD treatment, broader health care systems, and the child welfare and criminal justice systems. Recovery is a long-term process that may be initiated during treatment (one ecosystem), but will continue afterward, taking place primarily in the community (a second ecosystem). Ecosystems are themselves an important topic of research. A *Recovery Ready Ecosystem Model* and a *Recovery Ready Community Framework* (17) have been proposed to identify gaps in community infrastructure. Additionally, research utilizing a county-level recovery ecosystem index (REI) found that counties’ overdose death rates were inversely related to their REI score (18). A UK-based hub-and-spoke model designed to raise awareness of recovery and visibility of people in recovery may hold promise for building recovery infrastructure at the community level. Under this model, a non-profit organization operated three hubs offering an enriched array of services and supported and proactively built spoke networks through contracts, informal agreements, and ongoing outreach and engagement of key community partners. The spokes were built upon long-term relationships with individuals and communities and rely to a significant degree on volunteerism (19).

### Stigma

Stigma is pervasive, creating barriers to employment and housing, and is associated with reluctance to dispense buprenorphine among pharmacists (20), disruption of interpersonal relationships, harms to physical and mental health, and reduced help-seeking (21). The IWG highlighted the need for more intersectional research approaches that incorporate stigma as a critical factor that accounted for to more fully understand and support the recovery process. This includes research on the use of stigmatizing language about SUD and related topics, which remains prevalent in media, among health professionals, and elsewhere. Stigmatizing language is associated with greater attribution of blame to individuals for having an AOD disorder and with increased support for punitive responses (22).

## Discussion

While there is a need for additional recovery research, it must be balanced with other opportunities in addiction research. Because federal agencies identify research gaps independently, and have varying processes and timelines for doing so, there is a need for cross-agency coordination to advance recovery research. This will require alignment of processes with differing timelines and components across agencies. Similarly, agency resources for evaluating their own programs are limited and there may be statutory evaluation requirements that must be prioritized over other efforts. Data collection on recovery, whether in relation to annual surveys, services, epidemiological efforts, or programs, needs to be consistent from year-to-year. New elements or changes in survey questions or data elements must be weighed against the need to ensure comparability of annual data.

This mini-review article serves to mark progress made to date by the IWG. It may not be inclusive of all recovery research interests, including evaluation and data collection efforts that were launched after the completion of the IWG’s activities. In addition, research gaps may shift over time, affecting the research needed. Additional research is needed to improve our understanding of RSS, identify effective strategies for financing and organizing recovery-oriented systems of care, map the diverse pathways from SUD to recovery or remission, and validate recovery measures. Importantly, this research should strongly encourage the inclusion of PWLLE, utilize multi-level approaches assessing factors impacting recovery outcomes, and address stigma. This article offers a pathway forward for the growing field of recovery science to inform critical policy and resource allocation decisions with the goal of increasing the rates at which recovery and remission from SUD are initiated and sustained.
