# Supplementary material for: Substance use disorder recovery research opportunities: perspectives from a federal interagency workgroup
Source: Front Public Health. 2025 Apr 10;13:1585533. doi: 10.3389/fpubh.2025.1585533 (PMC12018379; doi:10.3389/fpubh.2025.1585533)
Supplement: Supplementary file 1 [file Data_Sheet_1.pdf]

Topic	Rationale / Notes	HHS/NIH/NIDA	HHS/NIH/NIAAA	HHS/AHRQ	HHS/ASPE	DoD	DOJ/BOP/SES	DOJ/OJP/NIJ	DOL/ODEP	DOL/ETA	DOL/OSHA	ED/IES	EOP/OSTP	HHS/CDC/OD/OC	HHS/CDC/NIOSH	HHS/CDC/NCIPC	HHS/CDC/OD/OS	HHS/CDC/OD/OC	HHS/CMS/CSSQ	HHS/CMS/CMMI	HHS/CDC/OD/NCHS	HHS/CMS/CMMS/CMCS	HHS/CMS/OFM	HHS/HRSA/OPAE	HHS/IHS	HHS/NIH/NIEHS	HHS/SAMHSA/OR	HHS/SAMHSA/CBHSQ	HUD/OPDR	VA/VHA
**Services**																														
**How effective are recovery support services, what is their cost ?** Recovery support services (RSS) include but are not limited to the following:		R	R	RED	RED	RED	RED	RED	RED	RED		RED	RE	RED	RED				RED	ED	RED		RED		ED		ED			RED
Recovery coaching?		R	R			RED	RED																RED		ED		ED			
Various other forms of peer recovery support services?		R	R																				RED		ED		ED			
Recovery housing *(various approaches)* ?		R	R																				RED		ED		ED		RE	
Recovery high schools?		R	R									RED													ED		ED			
Recovery Community Centers		R	R			RED																	RED		ED		ED			
Alternative Peer Groups?		R	R									RED											RED		ED		ED			
Other youth-focused services and supports?		R	R									RED													ED		ED			
Collegiate recovery programs?		R	R									RED													ED		ED			
Social and recreational models		R	R									RED													ED		ED			
Peer outreach and engagement (e.g., with overdose survivors in emergency departments or in the community, peer navigation)?		R	R													D							RED		ED		ED			
RCOs and PWLLE in provision of harm reduction services?		R	R																						ED		ED			
Existing recovery-supportive workplace programs, such as established recovery-friendly workplace programs and similar existing or developing programs.		R	R						RED	RED	RED														ED	RED	ED			
Recovery housing as a component of reentry							RED																							
Roles/models of peer support in diversion, reentry, and during incarceration							RED																							
Roles effectiveness of RCOs and other peer led organizations as recovery support service providers for individuals involved in the CJS.							RED																							
What services and approaches are effective for special-emphasis populations (e.g. co-occurring disorders ; Native Americans; Deaf Population; Disabilities, peole involved in CJS or child welfare systems, people with chronic pain and OUD, etc.)		R	R	RED	RED	RED	RED	RED	RED																ED		ED	ED		RED
**What data is available regarding the utilization and impact of different workplace resources related to SUD treatment and recovery (as in through EAP/MAPs)?**		R	R								RED					RED										RED		RED		
**What are the specific mediating and moderating factors that influence how RSS operate and work?**		R	R																											
**Are there components or mechanisms of RSS that are most effective , and are there differences for individuals with different needs and conditions (e.g. those with both substance use and co-occuring mental health conditions or other special-emphasis groups)?**		R	R																						ED		ED			
**Organization and Financing of Systems/Services**																														
What are the pros and cons of RSS delivery through peer-led organizations versus through treatment providers or other non-peer-led entities?		R	R	RED	RED			RED									RED													RED
How are RSS currently funded and what are the advantages and disadvantages of current approaches (including braided or blended funding, public and private insurance, grants, etc.)		R	R	RED	RED												RED											RED		
What reimbursement methods (e.g., bundled payments, fee-for-service, daily or weekly rates) best support efficient, effective, and sustainable service provision?		R	R	RED	RED												RED		RED		ED	ED						RED		RED
How are RCOs and peer workers linked to, coordinated with, or integrated into broader RSS networks (e.g., various forms of network analysis)?		R	R	RED	RED																									RED
How are RCOs and peer workers linked to, coordinated with, or integrated into the healthcare system (e.g., referrals, co-location, integration)?		R	R	RED	RED												RED													RED

Key

Key	
Research	R
Evaluation	E
Data	D
Research & Evaluation	RE
Research & Data	RD
Evaluation & Data	ED
Research, Evaluation & Data	RED

Note: This is an image of the first page of a four-page worksheet. It is intended to be illustrative and may not represent a comprehensive mapping of research, evaluation, or data collection efforts potentially within the scope of listed agencies. Additionally, this listing does represent a commitment by agencies to engage in research, evaluation or data collection in identified areas.

Supplement
