# Supplementary material for: Substance use disorder recovery research opportunities: perspectives from a federal interagency workgroup
Source: Front Public Health. 2025 Apr 10;13:1585533. doi: 10.3389/fpubh.2025.1585533 (PMC12018379; doi:10.3389/fpubh.2025.1585533)
Supplement: Supplementary file 2 [file Data_Sheet_2.pdf]

Supplement 2

Recovery Research Interagency Workgroup

Participating Agencies

Appalachian Regional Commission

Executive Office of the President

- Domestic Policy Council**
- Office of National Drug Control Policy*
- Office of Management and Budget**
- Office of Science and Technology Policy**

U.S. Department of Defense

- Health Affairs**
- Army Medical Research and Development Command*

U.S. Department of Education

- Institute of Education Sciences*
- Office of Elementary and Secondary Education***

U.S. Department of Health and Human Services

- Agency for Healthcare Research and Policy*
- Assistant Secretary for Planning and Evaluation*
- Centers for Disease Control and Prevention*
    - Division of Injury Prevention***
    - National Institute on Occupational Safety and Health***
- Centers for Medicare and Medicaid Services*
    - Center for Clinical Standards & Quality***
    - Center for Medicaid and Chip Services***
    - Data Analytics & Research Group***
    - Office of Financial Management***
    - Office of the Administrator***
- Health Resources and Services Administration*
- Indian Health Services*
- Office of the Secretary**
- National Institutes for Health
    - National Institute on Alcohol Abuse and Alcoholism*
    - National Institute of Environmental Health Sciences***
    - National Institute on Drug Abuse*

Substance Abuse and Mental Health Services Administration*

U.S. Department of Housing and Urban Development

Office of Policy Development and Research*

U.S. Department of Justice

Bureau of Prisons***

National Institute of Corrections*

Office of Justice Programs*

U.S. Department of Labor

Employment and Training Administration*

U.S. Department of Veterans Affairs

Veterans Health Administration*

---

* Participant designated in National Drug Control Strategy

** Voluntary Observer

*** Voluntary Participant
